# Investigation on the Thermal Conductivity of Mineral Oil-Based Alumina/Aluminum Nitride Nanofluids

**DOI:** 10.3390/ma12244217

**Published:** 2019-12-16

**Authors:** Dong Xiang, Liangping Shen, Hanbin Wang

**Affiliations:** 1 School of Mechatronics Engineering, Harbin Institute of Technology, Harbin 150001, China; xiangdong@hit.edu.cn; 2 Key Laboratory of Ferro and Piezoelectric Materials and Devices of Hubei Province, Faculty of Physics and Electronic Science, Hubei University, Wuhan 430062, China

**Keywords:** oil-based nanofluids, thermal conductivity, theoretical models

## Abstract

Al_2_O_3_/AlN–mineral oil nanofluids were prepared by dispersing commercially available Al_2_O_3_ and AlN nanoparticles into mineral oil. SEM measurements showed that the average diameter of the Al_2_O_3_ and AlN nanoparticles was about 55 and 50 nm, respectively. The experiments showed that the thermal conductivity systematically improved as the Al_2_O_3_ and AlN nanoparticles were introduced into the mineral oil. The thermal conductivity of the mineral oil-based nanofluids increased by 18% with a 1% volume fraction of Al_2_O_3_ and increased by 7% with a 0.5% volume fraction of AlN. The experimental data were compared with the values that were predicted by four typical thermal conductivity models, and a large disparity was disclosed between the models and the experimental data. After considering the thermal dynamic factors in the Al_2_O_3_/AlN–mineral oil nanofluids, a universal model is proposed that agrees well with the variation of thermal conductivity of the nanofluids.

## 1. Introduction

Due to the growing power output of engines and the miniaturization of microelectronic devices and ultra-high voltage power equipment in industries, traditional heat transfer fluids like mineral oil, water, and ethylene cannot satisfy recent high thermal transfer demands. It is therefore imminent to develop new fluids with better thermal conductivity than conventional ones. Recently, nanotechnology, such as micro- and nano-electronic technology and heat transfer enhancement, has rapidly developed and has been widely applied to industrial fields. It has been demonstrated that a fluid that contains suspended metallic or non-metallic nanoparticles possesses higher thermal conductivity than base fluids; this fluid was termed “nanofluid” by Choi et al. [1,2,3]. Since their introduction, nanofluids have shown great potential in many industrial fields to many industrial fields, such as electronics, nuclear reactors coolants, the space industry, and refrigeration.

Al_2_O_3_ and AlN materials are non-toxic and chemically stable materials with high thermal conductivity and mechanical strength. It is expected that dispersing nanosized Al_2_O_3_ or AlN into base fluids may result in considerable enhancements to thermal conductivity [4,5,6,7,8,9,10]. Some studies have been performed on Al_2_O_3_ and AlN nanoparticles that were dispersed in different hydrophilic fluids. For example, Lee at al. prepared Al_2_O_3_/ethylene glycol (EG) and Al_2_O_3_/water nanofluids with a particle size of 38 nm. The thermal conductivity of the nanofluids was increased by 11% with a 4.3 vol.% of Al_2_O_3_/water and by 19% with a 5 vol.% of Al_2_O_3_/ethylene glycol [11]. The thermal conductivity of Al_2_O_3_/water nanofluids was also studied by Putra et al., who showed that thermal conductivity enhancement reached 24.3% with a 4 vol.% of Al_2_O_3_ nanoparticles at 51 °C [12]. Noghrehabadi et al. experimentally investigated the convective heat transfer of a γ-Al_2_O_3_–water nanofluid in a circular tube; it was shown that the average heat transfer coefficient was increased by 16.8% with a 0.9 vol.% of the Al_2_O_3_–water nanofluid compared to distilled water [13]. As for AlN-nanofluids, Gaweł Zyła et al. prepared AlN/EG nanofluids with a particle size of around 50 nm [14]. They found that the thermal conductivity of the nanofluids was proportional to the volume fraction of AlN particles, and the highest thermal conductivity of 21% was achieved with the 7.9 vol.%. M. Wozniak et al. prepared AlN-propylene glycol (PPG) nanofluids and disclosed that the thermal conductivity increased with the volume fraction of AlN in PPG but had no relationship with temperature [15]. Yu et al. prepared two kinds of nanofluids by dispersing AlN nanoparticles in EG and PPG, and the enhancements of thermal conductivity were measured to be 38.71% and 40.2% with a 10.0 vol.% of AlN [16], respectively.

Though researchers have almost exclusively studied the thermal properties of glycol- or water-based Al_2_O_3_/AlN nanofluids, there has been limited research on oil-based Al_2_O_3_/AlN nanofluids. Notably, mineral oils have wide application in high voltage power equipment, and the development of mineral oil-based nanofluids might satisfy some special requirements in practical applications [17,18,19]. For this work, the thermal conductivity of Al_2_O_3_/AlN–mineral oil was studied as a function of the volume fraction of the nanoparticles. A deviation is revealed by comparing the experimental results with the values predicted by four typical thermal conductivity models. After considering both the static and thermal dynamic factors in the Al_2_O_3_/AlN–mineral oil nanofluids (referred to as Al_2_O_3_/AlN nanofluids), a universal model is proposed that agrees well with the variation of thermal conductivity of the nanofluids.

## 2. Materials and Methods

Al_2_O_3_/AlN–mineral oil nanofluids were prepared by dispersing commercially-purchased Al_2_O_3_ and AlN nanoparticles (Aladdin Reagent Inc., Shanghai, China.) into mineral oil. The average size of the γ-Al_2_O_3_ nanoparticles was about 55 nm, with a purity of 99 wt.%. The average size of the AlN nanoparticles was 50 nm, with a reagent purity of 99.5 wt.%. For the preparation of the nanofluids, 10 g of γ-Al_2_O_3_/AlN nanoparticles were deagglomerated by milling for 5 h. Then, the milled Al_2_O_3_ or AlN particles were put into a quartz boat, and 5 mL oleic acid was added. The mixture was milled again for 30 min. After milling, 50 mL of ethanol was added to the mixture, and the solution was ultrasonically vibrated for 30 min under a power of 200 W. The treated nanoparticles were centrifuged and were finally dispersed into mineral oil by stirring and ultrasonic vibration.

### Material Characterization

The morphology and microstructure of the γ-Al_2_O_3_/AlN nanoparticles were analyzed by using scanning electron microscopy (SEM, JEOL7100F, JSM, Tokyo, Japan). The thermal conductivity of modified mineral oil was measured by a heat conductivity meter (KD2-Pro, DECAGON, Pullman, WA, USA). The measurement of the KD2-Pro meter is based on the transient hot wire (THW) method and has a measurement accuracy of ±5%. During the measurement, the thin wire immersed in the nanofluid works not only as a thermal source but also as a temperature sensor. The thermal conductivity of the nanofluid can be calculated by [20]

(1) $$k=\;\frac{q}{4\pi \Delta T}\ln\frac{t_{1}}{t_{2}}$$

where *q* is the electric power of the wire equal to the thermal power (per unit wire length) and Δ*T* is the temperature differenced during the measured time *t*_1_–*t*_2_.

## 3. Results and Discussion

**Figure 1:**
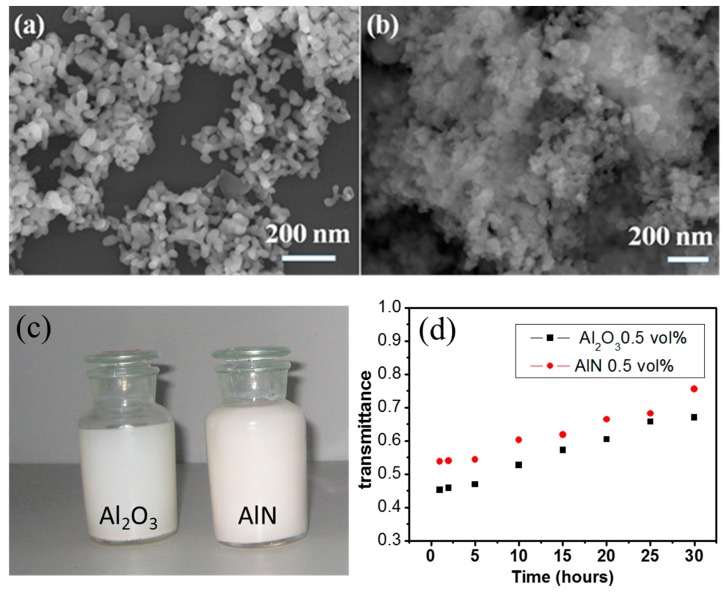
SEM images of (**a**) Al_2_O_3_ nanoparticles and (**b**) AlN nanoparticles. (**c**) Optical image of the Al_2_O_3_ and AlN nanofluids. (**d**) The transmittance of the nanofluids vs. sediment time.

Figure 1a,b shows SEM images of the Al_2_O_3_ and AlN nanoparticles loaded on the Si substrate. An optical image of the Al_2_O_3_ and AlN nanofluids is shown in Figure 1c. As displayed in Figure 1a, the Al_2_O_3_ nanoparticles had a smooth surface with a size ranging from 30 to 70 nm, and the average diameter was observed to be about 55 nm. As shown in Figure 1b, the milled AlN nanoparticles had a rough surface, and their average size was determined to be 50 nm with a wide size distribution. In this study, the thermal conductivity of the Al_2_O_3_/AlN–mineral oil nanofluids was measured with different volume fractions of nanoparticles at room temperature. The thermal conductivity of the Al_2_O_3_ and AlN nanoparticles was about 40 and 160 Wm^−1^K^−1^, respectively, which was much higher than that of the pure mineral oil (the measured value was 0.11 Wm^−1^K^−1^ at room temperature). It was found that the mineral oil-based nanofluids with a higher volume fraction (>1% for Al_2_O_3_ and >0.5% for AlN) easily aggregated from oil. The volume fractions of the Al_2_O_3_ and AlN nanoparticles in mineral oil were kept within 1.0% and 0.5%, respectively. The stability of the Al_2_O_3_ and AlN nanofluids was investigated by the UV-visible optical transmittance method. As shown in Figure 1d, the 0.5 vol.% Al_2_O_3_ and AlN nanofluids were quite stable for the first 5 h and then gradually precipitated from the mineral oil. This reflects that some of the Al_2_O_3_ and AlN particles still aggregated due to interactions between particles. In order to ensure the stability of the nanofluids during the measurement, the thermal conductivity data were recorded once the fresh nanofluids were prepared.

**Figure 2:**
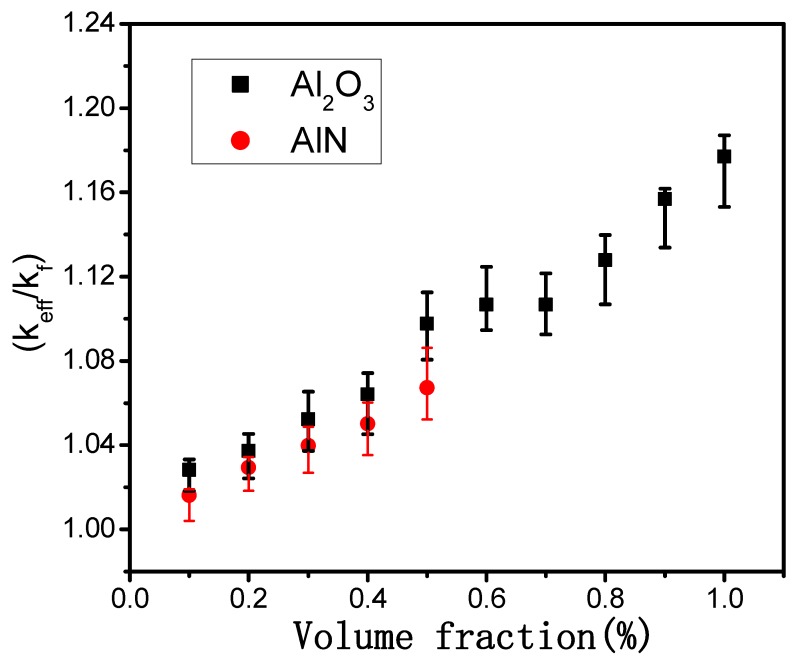
Thermal conductivity of the Al_2_O_3_/AlN–mineral oil nanofluids as a function of the volume fraction of nanoparticles.

Figure 2 plots the change of thermal conductivity of the Al_2_O_3_–mineral oil and AlN–mineral oil nanofluids as a function of the volume fraction of nanoparticles. Here, *k_eff_* is the effective thermal conductivity of the Al_2_O_3_/AlN nanofluids and the *k_f_* is the thermal conductivity of the pure mineral oil (0.1056 Wm^−1^K^−1^ at room temperature). The results showed that the thermal conductivity systematically improved as the Al_2_O_3_ and AlN nanoparticles were introduced into the base oil. The thermal conductivity of the modified mineral oil increased by 18% with the 1 vol.% of Al_2_O_3_ nanoparticles, and it increased by 7% with the 0.5 vol.% of AlN nanoparticles as well. It is worth noting that although the thermal conductivity of AlN nanoparticles was much higher than that of the Al_2_O_3_ nanoparticles, the thermal conductivity of the AlN–mineral oil was slightly smaller than that of the Al_2_O_3_–mineral oil nanofluids when they had an equal volume fraction. This suggests that the thermal conductivity of the added nanoparticles was not the key factor in determining the enhancement of the heat transfer in the nanofluids. These phenomena were likely due to the following reasons. (1) As revealed in a previous theoretical model, the Brownian motion of particles is the main factor that leads to variations of thermal conductivity; as both Al_2_O_3_ and AlN nanoparticles had a similar size, the effects of Brownian motion were roughly the same. (2) A surface stabilized layer (oleic acid) was adsorbed on the AlN/Al_2_O_3_ nanoparticles, and this would have inevitably complemented the impact of the thermal conductivity of nanoparticles on the thermal conductivity of the nanofluids.

As indicated in Figure 2, the thermal conductivity of the mineral oil was obviously enhanced when the volume fraction of nanoparticles was lower. For example, the thermal conductivity of the mineral oil-based nanofluids increased by 18% with the 1% volume fraction of Al_2_O_3_ and increased by 7% with the 0.5% volume fraction of AlN. These values are higher than those of water- or glycol-based Al_2_O_3_/AlN nanofluids with the same volume fraction [11,12,13,14,15,16]. In order to study the physical mechanisms associated with the oil-based nanofluids, the experimental values were compared with classical theoretical models. 

The Maxwell model is based on the assumption that spherical particles in liquid are uniformly suspended and do not aggregate. It presents the following dependence of conductivity of suspension on the volume fraction of the solid phase, which can be expressed as [21]:

(2) $$\frac{k_{eff}}{k_{f}}=\frac{k_{p}+2k_{f}-2\phi (k_{f}-k_{p})}{k_{p}+2k_{f}+\phi (k_{f}-k_{p})}$$

where *k_eff_* is the thermal conductivity of nanoparticle-modified mineral oil, *k_f_* is the thermal conductivity of pure insulation oil, *Φ* is the volume fraction of nanoparticles in nanofluids, and *K_p_* is the thermal conductivity of nanoparticles. 

Then, Hamilton and Crosser (HC) assumed that the heat transfer process between liquid and solid particles happens on the interface [22], and, as a result, the shape of particles influences the thermal conductivity of nanofluids. The thermal conductivity of the proposed nanofluid can be expressed as:

(3) $$\frac{k_{eff}}{k_{f}}=\frac{k_{p}+(n-1)k_{f}-(n-1)\phi (k_{f}-k_{p})}{k_{p}+(n-1)k_{f}+\phi (k_{f}-k_{p})}$$

where *n* is the empirical shape factor given by *n* = 3/ψ and ψ is the particle sphericity. 

It is supposed that liquid molecules around a solid surface often behave as layered solid-like structures, and, based on this, Yu and Choi deduced that this nanolayer that is attached on the solid phase has a close relationship to the thermal properties of suspensions [23]. They modified the Maxwell equation by considering the effects of the adsorption layer on the thermal conductivity of solid–liquid suspensions. It is of special note that this ordered adsorption layer has a significant impact on the thermal conductivity of nanofluids when the particles are smaller than 10 nm. Accordingly, a modified model for the thermal conductivity of nanofluids was proposed:

(4) $$\frac{k_{eff}}{k_{f}}=\frac{k_{p}+2k_{f}+2\phi (k_{p}-k_{f}){\left(1+2L_{layer}/d_{p}\right)}^{3}}{k_{p}+2k_{f}-\phi (k_{p}-k_{f}){\left(1+2L_{layer}/d_{p}\right)}^{3}}$$

where *L_layer_* is the thickness of the absorption layer, which is about 1–3 nm in general, and *d_p_* is the average diameter of nanoparticles.

According to Brownian theory, the increase of energy transport through suspended nanoparticles enhances the thermal conductivity of nanofluids. Considering the Brownian motion of the nanoparticles, Xuan et al. established a new thermal conductivity model for nanofluids [24]:

(5) $$\frac{k_{eff}}{k_{f}}=\frac{k_{p}+2k_{f}-2\phi (k_{f}-k_{p})}{k_{p}+2k_{f}+\phi (k_{f}-k_{p})}+\frac{\rho_{p}\phi C_{p}}{2k_{f}}\sqrt{\frac{k_{b}T}{3\pi r_{}\eta }}$$

where *ρ_p_* is the mass density of nanoparticles, *C_p_* is specific heat of nanoparticles, *k_b_* is the Boltzmann constant, *r* is the average radius of nanoparticles, *T* is the temperature, and *η* is the dynamic viscosity of the nanofluids. 

**Figure 3:**
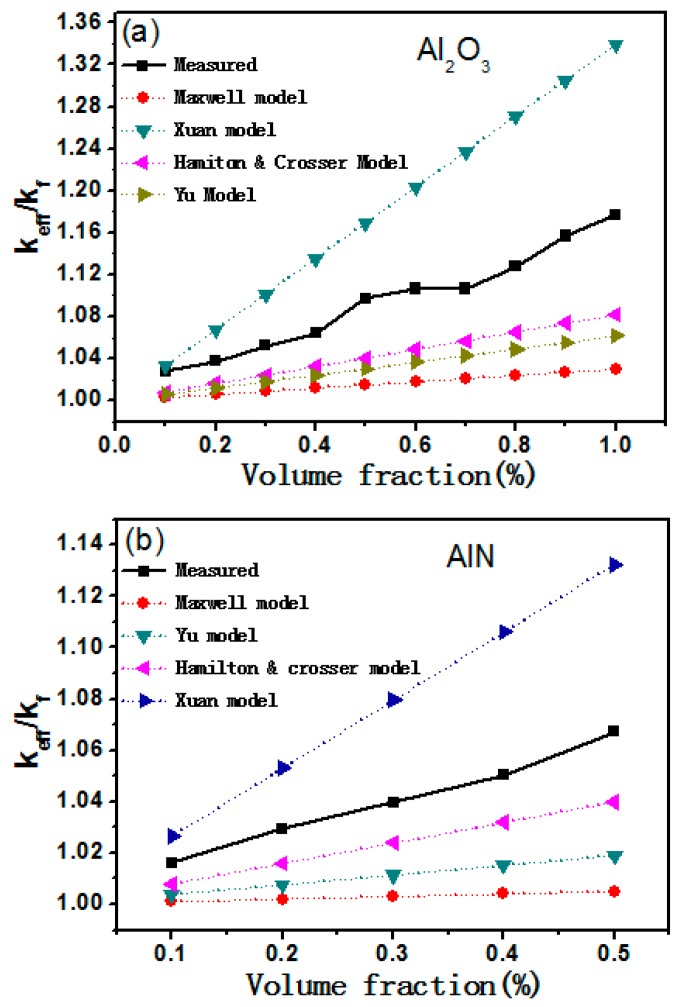
The measured thermal conductivity of (**a**) Al_2_O_3_–mineral oil nanofluids and (**b**) AlN–mineral oil nanofluids with theoretical values predicted by four classical models.

The average measured values of thermal conductivity of the Al_2_O_3_/AlN nanofluids were compared with the values predicted by the above four models. As plotted in Figure 3a,b, the values predicted by the Maxwell model had the maximal deviation from the experiment data. This indicates that the actual nanofluids were far from an ideal dispersion system. Similarly, the thermal conductivity predicted by the HC and Yu models was systematically smaller than the experiment data, although both particle sphericity and absorption layer were considered in these models. To simulate the improved thermal transfer process of the nanofluids, the dynamic factors such as the Brownian motion of nanoparticles should be considered. As also revealed in Figure 3, by considering Brownian motion, the Xuan model gave higher theoretical values than those predicted by the Yu or HC models. However, all the values predicted by the Xuan model were higher than the experimental data of the Al_2_O_3_– and AlN–mineral oil nanofluids. This suggests that was a deviation between the theoretical model and the actual situation of the Al_2_O_3_/AlN–mineral oil nanofluids. 

According to Brownian theory, the thermal transfer process between the nanoparticles contributed to the thermal conductivity of the nanofluids. The smaller the colloidal particles, the more intense their movement. Therefore, the energy transport inside the liquid became stronger as the size of the particles decreased. In the Xuan model, a nanofluid is regarded as dispersive system, and the size of single particle is taken as the average radius. In fact, the effective average radius of nanoparticles is higher than the radius of a single particle because many clusters easily form in nanofluids. These clusters often contain several nanoparticles and move slower than a single nanoparticle. Moreover, the growing clusters may aggregate under the gravity if the effect of gravity is greater than the Brownian motion. As a consequence, the contribution of thermal transport through nanoparticles decreases in nanofluids. 

In nanofluids, dynamic viscosity is a function of the volumetric fraction of nanoparticles. Generally, there is a positive correlation between dynamic viscosity and volume fraction. When the concentration of a nanofluid is lower than 10 vol.%, the dependence of the volumetric fraction of the nanofluids on the dynamic viscosity is given by Equation (6) [25],

(6) $$\eta =\eta_{f}(1+2.5\phi +6.25\phi^{2})$$

where *η* is the effective dynamic viscosity of the nanofluid and *η_f_* is the dynamic viscosity of the pure base fluid.

Finally, a universal thermal conductivity model for the Al_2_O_3_/AlN–mineral oil nanofluids can be deduced by considering both static and dynamic factors such as Brownian motion, particle aggregation, and dynamic viscosity variation. The new thermal conductivity model is defined as:

(7) $$\frac{k_{eff}}{k_{f}}=\frac{k_{p}+\;(\phi_{s}\rho_{p}r-1)k_{f}-(\phi_{s}\rho_{p}r-1)\phi (k_{f}-k_{p}){\left(1+L_{layer}/r\right)}^{3}}{k_{p}+(\phi_{s}\rho_{p}r-1)k_{f}+\phi (k_{f}-k_{p}){\left(1+L_{layer}/r\right)}^{3}}+\frac{\rho_{p}\phi C_{p}}{2k_{f}}\sqrt{\frac{k_{b}T}{3\pi r_{c}\eta_{0}(1+2.5\phi +6.25\phi^{2})}}$$

where *ρ_p_* is the mass density of nanoparticles, *Φ_s_* is the specific surface of nanoparticles, *Φ* is the volume fraction of nanoparticles in nanofluids, *η*_o_ is the effective dynamic viscosity of the pure oil at temperature *T*, *r_c_* is the average radius of clusters, *C_p_* is specific heat of nanoparticles, and *k_b_* is the Boltzmann constant. 

**Figure 4:**
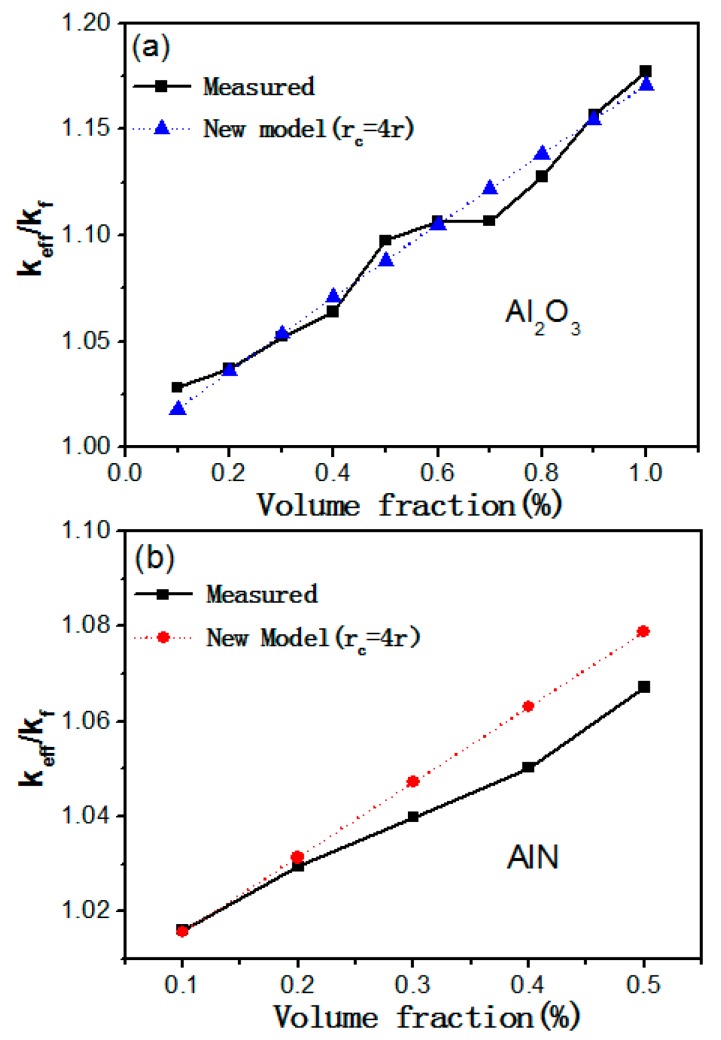
New model that validates the thermal conductivity of (**a**) Al_2_O_3_–mineral oil nanofluids (**b**) AlN–mineral oil nanofluids as a function of volume fraction.

In order to validate the effectiveness of the new model, the thermal conductivity calculated by the new model and the average experiment data are plotted in Figure 4. As shown in Figure 4, the calculated values were in good agreement with the experiment data when the average radius of the clusters was taken as four times of the single particle. The calculated values of the Al_2_O_3_–mineral oil nanofluids agreed well with measured data in Figure 4a. A moderate deviation between theoretical values and experimental data can be observed In Figure 4b for the AlN–mineral oil nanofluids, especially in the higher volume fraction range. Such difference were probably due to the aggregation of the AlN nanoparticles in the mineral oil. In the new model, the dispersity of nanoparticles in the nanofluid is one of the key factors that determines the thermodynamic properties of the nanofluid. As displayed in Figure 1d, the AlN nanoparticles showed an inferior dispersity than the Al_2_O_3_ nanoparticles in the mineral oil. Such aggregation was more obvious at the higher volume fraction of AlN, and it therefore caused a deviation between the theory and experimental values. In conclusion, by considering the thermodynamic properties of nanofluids such as particle aggregation and dynamic viscosity variation, the proposed model is effective in describing the variation of the thermal conductivity of Al_2_O_3_/AlN mineral oil nanofluids.

## 4. Conclusions

In summary, the thermal conductivity of Al_2_O_3_/AlN–mineral oil nanofluids was investigated as a function of the volume fraction of Al_2_O_3_/AlN nanoparticles. The nanofluids showed an improved thermal conductivity in comparison with the pure mineral oil. The thermal conductivity variation of the Al_2_O_3_/AlN–mineral oil did not follow traditional models such as the Maxwell or HC models. A new model was established by considering both the static factors and the dynamic factors such as Brownian motion, particle aggregation, and dynamic viscosity variation. The new model is effective in describing the variation of the thermal conductivity of Al_2_O_3_/AlN mineral oil nanofluids.
